# Clinical Importance of Placental Testing among Suspected Cases of Congenital Zika Syndrome

**DOI:** 10.3390/ijms20030712

**Published:** 2019-02-07

**Authors:** Maxim D. Seferovic, Michelle Turley, Gregory C. Valentine, Martha Rac, Eumenia C. C. Castro, Angela M. Major, Brianna Sanchez, Catherine Eppes, Magdalena Sanz-Cortes, James Dunn, Tiffany F. Kautz, James Versalovic, Kenneth L. Muldrew, Timothy Stout, Michael A. Belfort, Gail Demmler-Harrison, Kjersti M. Aagaard

**Affiliations:** 1 Departments of Obstetrics & Gynecology, Division of Maternal-Fetal Medicine, Baylor College of Medicine, Houston, TX 77030, USA; maxim.seferovic@bcm.edu (M.D.S.); michelle.turley@bcm.edu (M.T.); gvalenti@bcm.edu (G.C.V.); martha.rac@bcm.edu (M.R.); brianna.sanchez@bcm.edu (B.S.); catherine.eppes@bcm.edu (C.E.); magdalena.sanzcortes@bcm.edu (M.S.-C.); tiffany.kautz@bcm.edu (T.F.K.); belfort@bcm.edu (M.A.B.); 2 Pediatrics, Section of Neonatology, Baylor College of Medicine, Houston, TX 77030, USA; 3 Pathology and Immunology, Baylor College of Medicine, Houston, TX 77030, USA; ecastro@bcm.edu (E.C.C.C.); amajor@bcm.edu (A.M.M.); jjdunn@texaschildrens.org (J.D.); jamesv@bcm.edu (J.V.); kenneth.muldrew@bcm.edu (K.L.M.); 4 Microbiology and Molecular Virology, Baylor College of Medicine, Houston, TX 77030, USA; 5 Ophthalmology, Baylor College of Medicine, Houston, TX 77030, USA; tim.stout@bcm.edu; 6 Pediatrics, Section of Infectious Diseases at Baylor College of Medicine and Texas Children’s Hospital, Houston, TX 77030, USA; gdemmler@bcm.edu; 7 Molecular and Human Genetics, Baylor College of Medicine, Houston, TX 77030, USA

**Keywords:** arbovirus, Zika virus, congenital Zika syndrome, placental Zika infection, microcephaly, congenital brain malformations, molecular virology, viral pathogenicity, placental testing

## Abstract

Contemporaneous Zika virus (ZIKV) strains can cause congenital Zika syndrome (CZS). Current ZIKV clinical laboratory testing strategies are limited and include IgM serology (which may wane 12 weeks after initial exposure) and nucleic acid testing (NAT) of maternal serum, urine, and placenta for (+) strand ZIKV RNA (which is often transient). The objectives of this study were to determine if use of additional molecular tools, such as quantitative PCR and microscopy, would add to the diagnostic value of current standard placental ZIKV testing in cases with maternal endemic exposure and indeterminate testing. ZIKV RNA was quantified from dissected sections of placental villi, chorioamnion sections, and full cross-sections of umbilical cord in all cases examined. Quantitation with high-resolution automated electrophoresis determined relative amounts of precisely verified ZIKV (74-nt amplicons). In order to localize and visualize stable and actively replicating placental ZIKV in situ, labeling of flaviviridae glycoprotein, RNA ISH against both (+) and (−) ZIKV-specific ssRNA strands, and independent histologic examination for significant pathologic changes were employed. We demonstrate that the use of these molecular tools added to the diagnostic value of placental ZIKV testing among suspected cases of congenital Zika syndrome with poorly ascribed maternal endemic exposure.

## 1. Introduction

Zika virus (ZIKV) is a mosquito-borne (*Aedes genus*) arbovirus (arthropod-borne virus) of the *Flaviviridae* family. Originally discovered in Uganda in 1947 [1], initial outbreaks of ZIKV were largely sporadic across Southeast Asia and Africa. As the virus spread eastward, Yap Island became endemic in 2007, followed by epidemics in French Polynesia, New Caledonia, the Cook Islands, and Easter Island in 2013 and 2014 [1,2]. By 2014, ZIKV had reached the Americas with the initial outbreaks occurring in the Caribbean and South America and then broadening to include vast swathes across the Western Hemisphere as of late 2018 (https://www.cdc.gov/zika/geo/index.html) (accessed on 1 December 2018). The virus is geographically spread as a result of human travel from endemic regions, alongside human-to-human transmission via sexual intercourse, blood transfusions, and via vertical maternal-fetal transmission [2,3,4,5]. Although no other flavivirus is known to cause disseminated fetal neural malformations in humans, worldwide concern for latent viral disease was raised following several case reports demonstrating persistent ZIKV RNA in the amniotic fluid, placenta, and fetal neural tissue weeks to months after initial maternal infection [3,4].

The positive-sense, single-stranded RNA genome of Zika virus encodes 10 genes, which are categorized as structural (capsid, premembrane, and envelope) or nonstructural (NS1, NS2A, NS2B, NS3, NS4A, NS4B, and NS5). Like other flaviviruses, the structural proteins form the outer barrier of the virus, while the nonstructural proteins are necessary for virus genome replication, immune evasion, and protein processing [2]. After attachment to a host cell using a debated receptor [6,7], the virus enters the cell via clathrin-mediated endocytosis [8]. Following fusion of a lysosome to the endosome containing the virion, the resulting drop in pH causes a conformational shift in the virus structure resulting in the deposit of the virus genome into the cytoplasm where it can be translated as a polyprotein by host ribosomes [2]. Once the genome is replicated by the nonstructural proteins, capsid proteins surround the virus genome to form an icosahedral structure, which travels as a nucleocapsid through the endoplasmic reticulum to become coated in premembrane and envelope proteins. During transport through the Golgi apparatus to the cell surface, a host furin protease cleaves the premembrane proteins to finalize virion maturation, finally resulting in the release of the virion from the plasma membrane [2].

ZIKV can infect many cell types [9,10,11,12,13,14,15,16,17,18,19,20,21,22,23,24,25,26,27,28,29,30,31,32], but in adult human and non-human primates it primarily targets ocular and reproductive tissues [33] (i.e., germ and somatic cells in the testes [9] in men and uterine fibroblasts [10] in women) and has been isolated from body fluids, such as blood [11], tears [12], sperm [13], and vaginal fluids [14]. In pregnant women, Hofbauer, trophoblast, and endothelial cells in the placenta have also been shown to be susceptible to ZIKV infection and serve as a reservoir [24,28,31,32,34,35,36,37,38]. It is presumed that from this reservoir there is vertical transmission, leading to ZIKV infection in the developing fetus where ZIKV preferentially infects neural progenitor cells [15], although neurons and astrocytes can also become infected [16].

In the United States and elsewhere, many of our institutions and clinical settings are challenged with the realities of prenatal care delivery among an under-resourced population at-risk for endemic ZIKV exposure and delayed entry to care. Although the WHO and CDC note decreasing prevalence of Zika virus (ZIKV) infection cases in the Americas (inclusive of North American, Central American, and South American countries and territories; [17]), it remains an ongoing clinical problem of significant magnitude for women and their providers in endemic and non-endemic, but nearby, regions. For example, in Texas, the second largest birth populous in the U.S. and the only state with ongoing local transmission, 219 pregnant women with laboratory evidence of possible recent ZIKV infection were reported from January 2016 to July 2017 [18]. Due to limitations and underutilization of current laboratory testing strategies for ZIKV, this number likely underestimates the number of cases with ZIKV infection in pregnancy by 57% or more [18]. For example, given the transient nature of ZIKV RNA, laboratory based nucleic acid testing (NAT) of serum, urine, and amniotic fluid does not exclude prior infection. Similarly, accurate interpretation of negative IgM serologic testing relies on timely serum collection: Collection before the development of IgM antibodies or after these antibodies have waned (two and 12 weeks, respectively) will lead to misinterpretation of negative results [17,19]. Conversely, positive IgM antibodies may represent other flavivirus infection or prior immunization [17,19]. Recognizing these limitations, the CDC recently updated its guidance for more limited testing of pregnant women with the goal of reducing false positive testing among gravidae with low pre-test probability. In doing so, they acknowledge the risk in delayed or missed identification of congenital Zika syndrome [17]. Importantly, a few reports have described negative or missed maternal testing in instances where infants were later found to have clinical findings consistent with possible congenital Zika syndrome [2,17,20,21,22]. 

As a result of these limitations in current standard clinical testing, there is an evident need for clinically useful and reliable alternative testing strategies—alone or in combination—to enable diagnosis of congenital Zika syndrome among women and their infants with ongoing risk but unclear timing of infection. In this report, we present four recent cases of gravidae who were at risk for endemic ZIKV infection preconception and/or during pregnancy, but with varying instances of delayed or indeterminate testing using current standard testing. Combining standard of care clinical prenatal laboratory testing and fetal imaging, three cases were deemed prenatally as having probable evidence of congenital Zika syndrome, while a fourth appeared phenotypically unaffected. However, NAT and RNA in situ hybridization (ISH) demonstrated evidence of placental infection among all three cases with a postnatal clinical diagnosis of congenital Zika syndrome. The fourth and clinically unaffected infant was negative by multiple measures. These data in our case series format collectively provide in situ evidence in humans for ongoing transplacental and paraplacental replication, consistent with the placenta functioning as a reservoir and route for fetal infection months after maternal clearing of the virus and loss of IgM seropositivity.

## 2. Results

### 2.1. Case Descriptions

**Figure 1:**
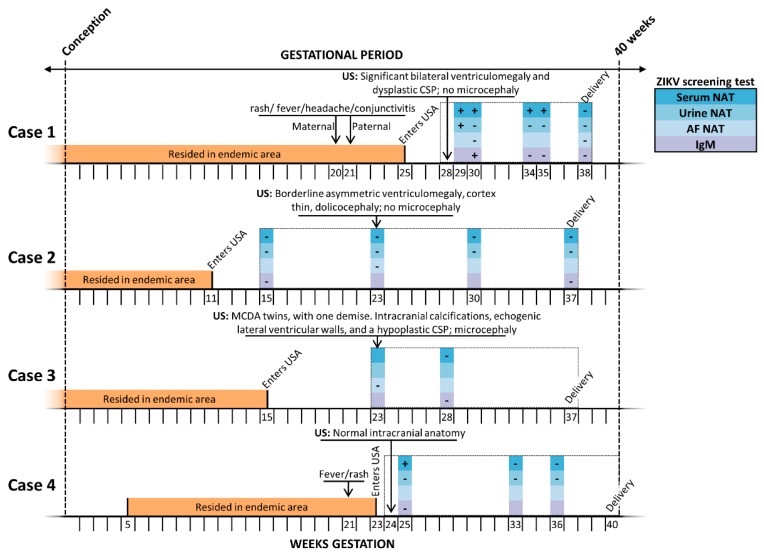
Timeline of subjects’ exposure risk, clinical findings, and standard clinical laboratory testing for ZIKV. The entire gestational interval during which antenatal care was delivered is outlined by a checked box, with annotated possible windows of endemic exposure (orange bar) and post-emigration ZIKV maternal screening (nucleic acid testing (NAT) or serologic IgM), colored by type and fluid tested (blue/purple legend). Estimated date of delivery (EDD, 40 weeks’ gestation; dashed line far right) was calculated based on obstetrical estimate, and confirmed by clinical best practice sonographic measures; interval week of gestational age is denoted on the X axis. Key findings by prenatal ultrasound (US) and/or MRI and gestational age at delivery are shown. CSP—cavum septum pellucidum; MCDA—monochorionic, diamniotic twin gestation; NAT—nucleic acid testing.

**Figure 2:**
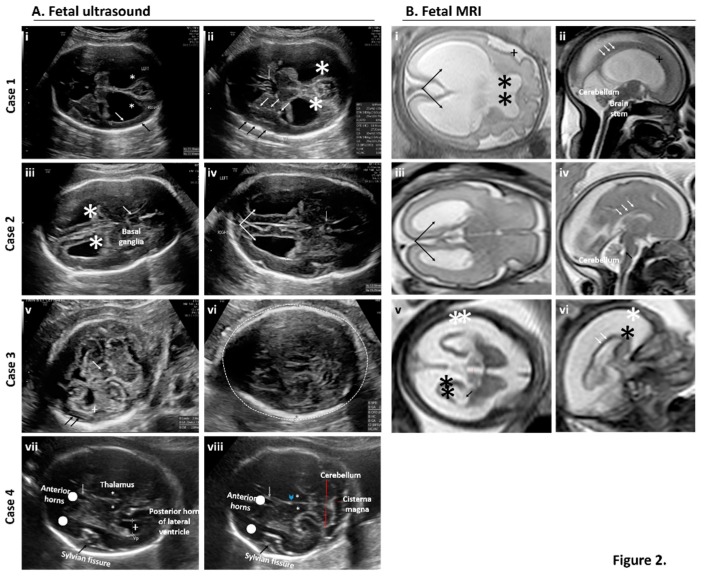
Prenatal examination of brain features by fetal neurosonography (**A**) and fetal MRI (**B**) for cases of suspected exposure to ZIKV. (**A**) Serial and repeat sonographic fetal imaging with neurosonography in the second and third trimester were performed. Case 1: Axial views of the fetal brain at 31 weeks and one day gestational age by best obstetrical estimate (31w1d). There is thinning of the cortex (white arrow) and reduced subarachnoid space (black arrow). Bilateral ventriculomegaly is present (star) (i). At the level of the thalami, (ii) punctiform calcifications are present within the basal ganglia (white arrows) and the subarachnoid space is reduced near the Sylvian fissure (black arrows). The cavum septum pellucidum is present but appears dysplastic (grey arrow). Bilateral ventriculomegaly is present (star). Case 2: Axial views of the fetal brain at 28w5d. Bilateral ventroculomegaly and mild colpocephaly is present (iii, star). The cavum septum pellucidum is present but dysplastic (iv, grey arrow). Bilateral colpocephaly appears asymmetric (iv, arrows). Case 3: Axial views of the fetal brain at 25w5d. There is thalamic hypoplasia (v, white arrow), severe cortical atrophy (v, cross), and a prominent subarachnoid space (v, black arrows). The posterior fossa is normal. (vi) Image is at the level of the transthalamic plane in which the biparietal diameter and head circumference is measured. Microcephaly (defined as head circumference <3SD) is present (dashed line). Case 4: Transthalamic (vii) and transcerebellar (viii) views at 29w3d. No obvious intracranial abnormalities are seen or suspected. Normal structures are labeled. Anterior horns (dots), cavum septum pellucidum (grey arrow), Sylvian fissure (black arrows), thalami (stars), and third ventricle (arrowhead). Cerebellum and cisterna magna (red lines). (**B**) Fetal MRI with axial views of the transthalamic plane and ventricular system alongside sagittal views of the cortex, cerebellum, brain stem, and corpus callosum revealed abnormalities in three of the four cases. Case 1 (i–ii): Axial and sagittal views at 31w1d. In addition to bilateral ventriculomegaly (i, arrows), there is an abnormal sulcation pattern seen in the Sylvian fissure (i and ii, cross). The leaflets of the cavum septum pellucidum were seen ventrally (not pictured) but not dorsally (i, stars). The corpus callosum was also thin near the splenium (ii, white arrows). The brain stem and cerebellum were normal. Case 2 (iii–iv): Axial and sagittal views at 28w5d. There was mild bilateral ventriculomegaly with a mild colpocephalic appearance (iii, black arrows). The corpus callosum is thin and only partially seen in the area of the splenium (iv, white arrows). The fourth ventricle is not dilated (iv, black arrows) and the brain stem and cerebellum were normal. Case 3 (v–vi): Axial and sagittal views at 25w5d. Severe cortical atrophy is present (v–vi, black stars) with massive enlargement of the subarachnoid space (v–vi, white stars). The lateral ventricles (v, black arrows) are mildly dilated, as is the third ventricle (v, red line). In the midsagittal plane, the corpus callosum is difficult to visualize (vi, white arrows). In real time, the brain matter “jiggled” with fetal movement due to severe atrophy and thinning (supplemental video). An MRI for Case 4 was not performed given the absence of sonographic findings by neurosonography.

**Figure 3:**
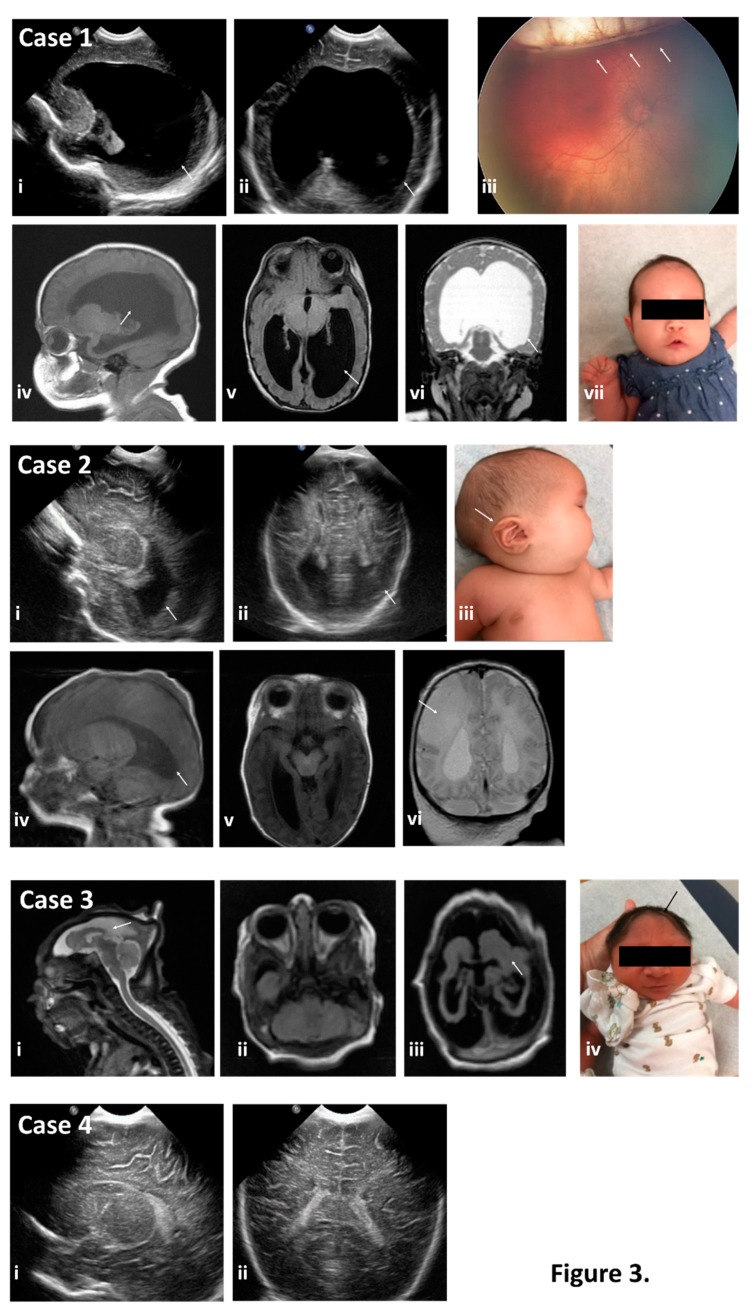
Neonatal physical exam findings consistent with a diagnosis of congenital Zika syndrome as identified by neonatal head ultrasound, postnatal MRI, gross presentation, and ophthalmic examination for the cases where detailed examination was warranted and subjects returned for postnatal care. Case 1 exhibited severe ventriculomegaly (arrows in images i and ii) by neonatal head ultrasound and as confirmed by post-natal MRI (arrows in images iv, v, and vi), despite the fronto-occipital head circumference (FOC) being normal (54th percentile) (image vii). Ophthalmic examination revealed bilateral colobomatous lesions consistent with congenital Zika syndrome (arrow in image iii). Case 2 exhibited colpocephaly of the lateral ventricles by head sonography (arrows in images i and ii), and postnatal MRI confirmed the colpocephaly (arrow in image iv) as well as callosal dysgenesis. Subject had low-set ears on exam (arrow in image iii) and a fronto-occipital circumference (FOC) at the 96th percentile with a cleft palate, microopthalmia, and asymmetric hypertonia with asymmetric EEG findings consistent with abnormalities in the right hemisphere. She developed neonatal seizures and failed her newborn hearing screen. Consistent with her clinical exam, she had findings concerning for an age-indeterminate vascular insult in the right middle cerebral artery territory with loss of gray-white differentiation and sulcal effacement within the right parietal lobe (arrow in image vi). Case 3 demonstrated significant cerebral dysplasia with severe microcephaly and supratentorial volume loss (arrows in images i, ii, and iii) on brain MRI. Physical exam also showed severe microcephaly (arrow in image iv) with FOC < −3.72 SD (<0.01%) per WHO growth curves. Case 4 exhibited no abnormal findings on postnatal head sonogram (images I and ii). Subject’s FOC was normal at the 36th percentile. No MRI in Case 4 was obtained given normalcy of physical exam and prenatal and postnatal sonographic findings.

A schematic summary of each case and representative fetal images are provided in Figure 1 and Figure 2, respectively. In Figure 1 a detailed timeline by weeks of gestation depicts the period of residence in a ZIKV endemic area, and timing of entry into the U.S., positive and negative maternal laboratory testing, and pertinent positive sonographic findings. All testing depicted in Figure 1 was performed in a clinical lab and thus depicts current standard testing, utilizing FDA approved ZIKV NAT (either RT-PCR or transcription mediated amplification, TMA) and IgM (MAC-ELISA) clinical assays. Figure 2 shows representative pre and postnatal ultrasound with neurosonography and MRI images. Key clinical aspects relative to the interpretation and findings of each case include:

Case 1: A 19-year-old previously healthy gravida 3 para 2002 emigrated to the U.S. at 25 weeks of gestation. During the 20th week of gestation, she and her family members (including her spouse) all experienced a maculopapular rash, conjunctivitis, fever, and headache; she and her spouse had unprotected intercourse through the first and second trimester. Approximately two weeks after their symptoms resolved, she and her spouse traveled by bus and foot across Honduras, Guatemala, and Mexico. At the time of initial presentation to care in the U.S. at 28 weeks’ gestation, she had positive ZIKV IgM serology and a positive serum NAT. Initial fetal ultrasound with neurosonography was significant for cerebral ventriculomegaly (20–25 mm) with dangling choroid, prominent 3rd ventricle, and a widened cavum septum pellucidum; microcephaly was never observed. An amniocentesis was performed, and showed a negative NAT for ZIKV with no evidence of small or large structural chromosomal variations by CMA; the TORCH panel was also negative. Repeat maternal serum testing for ZIKV by NAT was persistently positive until 38 weeks’ gestation, when she became NAT seronegative; at 34 weeks ZIKV serology (IgM) became negative. At 38 weeks and two days gestation (38w2d) an indicated cesarean was performed for oligohydramnios with fetal breech presentation. At delivery or postnatally, maternal and/or neonatal testing for ZIKV in serum, amniotic fluid, CSF (neonatal), and urine was negative. Neonatal and infant findings including ophthalmic exam and abnormal postnatal brain and head imaging are depicted in Figure 3. Key and persistent postnatal imaging findings include ventriculomegaly with absent cavum septum pellucidum, prominent third ventricle without obstruction at the level of the foramen of Monro, and diffuse white matter and corpus callosum volume loss with multiple sub-centimeter subependymal nodules along the lateral ventricles. On ophthalmic exam, the infant had bilateral colobomatous lesions consistent with congenital Zika syndrome. The infant has been readmitted on multiple occasions during the first six months of life for hypotonia and epileptic encephalopathy with infantile spasms and modified hypsarrhythmia requiring neuroleptic medications with global developmental delay. 

Case 2: A 24-year-old previously healthy gravida 3 para 0200 arrived in the U.S. at 11 weeks of gestation, after emigrating from Cuba through Central America and Mexico. She and her husband denied any symptoms consistent with a flavivirus infection; they had unprotected intercourse through the first and early second trimester. She was seen for her initial prenatal visit at 15 weeks’ gestation, and testing for ZIKV by serum and urine NAT and IgM were initially and persistently negative. A fetal ultrasound with neurosonography performed at 23 weeks showed no microcephaly but was significant for borderline asymmetric ventriculomegaly, with a thin cortex and dolichocephaly, but absence of microcephaly; amniocentesis was declined. Serial fetal ultrasound imaging throughout pregnancy was performed secondary to initial 23-week ultrasound findings. At 37 weeks, induction of labor was undertaken for intrahepatic cholestasis of pregnancy and a cesarean delivery was performed for fetal intolerance of labor. Neonatal findings were significant for craniofacial dimorphism with a cleft palate, microopthalmia, and hypertonia with asymmetric EEG findings consistent with abnormalities in the right hemisphere. She developed neonatal seizures and failed her newborn hearing screen. Representative neonatal and infant brain imaging are shown in Figure 3, and include callosal dysgenesis with asymmetric ventriculomegaly, a thin cortex, and dolichocephaly with bilateral optic nerve hypoplasia. There were no patellar nor limb abnormalities. Neonatal and infant ZIKV and TORCH laboratory testing was negative. No evidence of small nor large structural chromosomal variations by CMA was found, and whole exome sequencing (WES) failed to reveal neither pathogenic nor likely pathogenic variants in disease genes related to the clinical phenotype. A novel de novo heterozygous c.3667G>C (p.V1223L) variant in the *MED12* gene (located on ChrX:70349255) of unknown clinical significance (VUS) was found. This female infant has clinically persistent neurologic disease and early developmental delay.

Case 3: A 21-year-old previously healthy primigravid emigrated to the U.S. at 16 weeks’ gestation after two weeks of travel by bus and foot across El Salvador and Mexico. She and her husband denied any symptoms of flavivirus infection, and had unprotected intercourse through the first and early second trimester. After delayed entry into prenatal care, she presented for her initial prenatal sonogram at 23 weeks’ gestation and was noted to have a probable monochorionic, diamniotic twin gestation with demise of one twin at approximately 15 weeks. The surviving twin was found to be microcephalic with significant parenchymal volume loss, intracranial calcifications, and a hypoplastic cavum septum pellucidum. An amniocentesis was performed on fluid from the sac of the surviving twin and was negative for ZIKV NAT, toxoplasma, or CMV NAT, and there was no evidence of chromosomal aberrations on CMA; initial maternal serologic testing for ZIKV was negative at 28 weeks. The differential diagnoses included twin–twin transfusion syndrome versus congenital Zika syndrome with early fetal demise of one twin. She was delivered vaginally at 37 weeks via after a spontaneous labor. At delivery, the neonate had significant microcephaly and evidence of optic nerve atrophy on ophthalmic exam; neonatal serum and urine testing for ZIKV was negative, and representative neonatal imaging and clinical findings are shown in Figure 3. The infant has clinically persistent neurologic disease and early developmental delay.

Case 4: An 18-year-old previously healthy primigravid emigrated from Mexico to the U.S. at 23 weeks’ gestation. She reported a fever and rash approximately two weeks earlier. At 25 weeks’ gestation she presented for initial prenatal care. Her maternal ZIKV laboratory testing was positive for serum NAT but negative for IgM serologies. An initial fetal ultrasound including neurosonography was negative, as were follow up fetal sonograms. Repeat maternal NAT in serum and urine were persistently negative, as was neonatal testing. Follow up clinical examinations did not reveal any abnormal nor significant findings, and neither genetic testing nor neonatal brain imaging was clinically indicated. The infant showed appropriate neural development at the time of its pediatric well-child visit. 

### 2.2. Placental Testing

**Figure 4:**
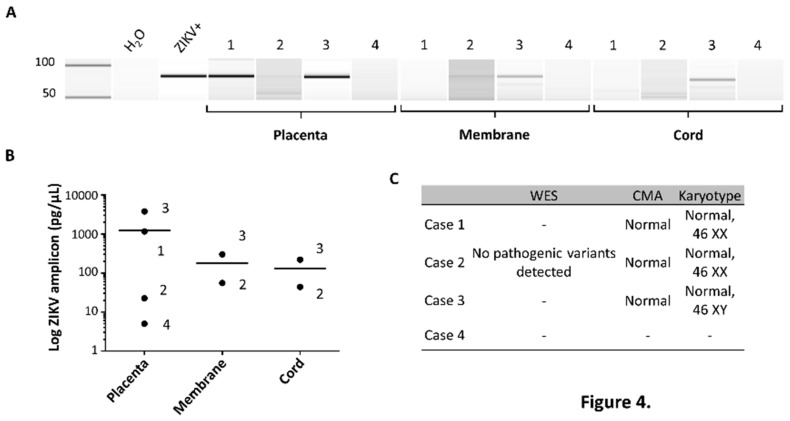
NAT-based assessment of non-formalin fixed placental, membrane, and/or umbilical cord tissue for ZIKV demonstrates detectable viral RNA in congenital Zika syndrome-affected cases, but not in the unaffected case. (**A**) ZIKV NAT in placental, membrane, and cord specimens. ZIKV was detected by NAT in all placental specimens in Cases 1, 2, and 3 (congenital Zika syndrome-affected) but not Case 4 (unaffected). ZIKV was detected in the membranes and cord of Cases 2 and 3 by NAT, but not Cases 1 nor 4. Heat killed conditioned media from Vero cells, with actively replicating clinical isolate ZIKV (20), served as a positive control (ZIKV+), and water as a negative control (H_2_O). (**B**) Quantitative bioanalysis band chromatograms from NAT. Quantitation of ZIKV (74 nt) in relative log measures by NAT was determined employing the Agilent BioAnalyzer. (**C**) Absence of detectable copy number or potentially pathologic SNP nor exon variants by CMA and whole exome sequencing. A novel de novo heterozygous c.3667G > C (p.V1223L) variant in the MED12 gene (located on ChrX:70349255) of unknown clinical significance (VUS) was found. No advanced genomic testing was performed with Case 4 secondary to absence of ultrasound findings and normal neonatal and infant clinical examinations.

**Figure 5:**
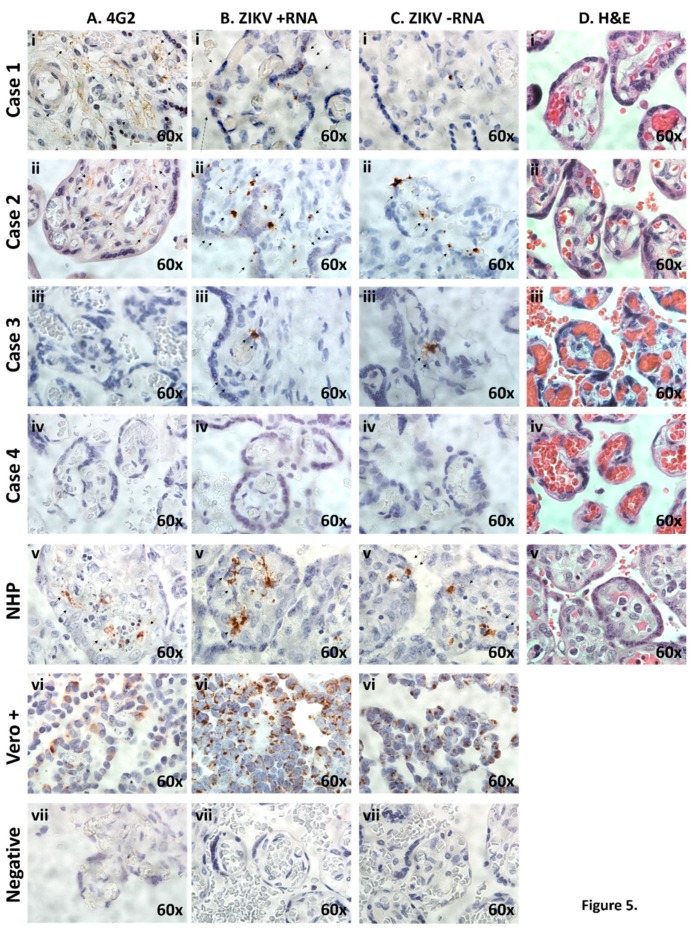
Histological and IHC examination of placentae reveals evidence of ZIKV infection and active placental replication in congenital Zika syndrome affected cases, but not unaffected. (**A**) Placental cross-sections were probed using the 4G2 antibody designed against flavivirus envelope protein(s). Diffuse villous and stromal labeling (brown staining, black arrows) were observed in congenital Zika syndrome affected Cases 1 and 2, inconclusive in Case 3, but with no labeling in unaffected Case 4. (**B**,**C**) In situ hybridization with oligo probes against plus (+; B) and minus (−; C) ssRNA ZIKV strands were directly assessed with excitation microscopy. Single molecule ssRNA ISH, employing stably amplified ZIKV probes, as previously described [19], was utilized, with summarily positive hybridization (brown labeling, black arrows) in congenital Zika syndrome affected (Cases 1–3) but not in the unaffectedcase (Case 4). In situ ZIKV (+) and (−) strand hybridization was used to distinguish between stable viral strand replicons (plus ssRNA strand, B) and actively replicating (minus strand ssRNA) of the ZIKV genomic replicon, as previously described [19]. (**D**) H&E-tained sections of placental villus and parenchyma showed no evidence of inflammation in neither congenital Zika syndrome affected nor unaffected cases. No histologic evidence of inflammation was observed with ZIKV infection in any of the human or NHP tissue examined, consistent with previous reports (panel **D**). In all microscopy experiments shown in panels **A**–**D**, comparison with an in vivo infected non-human primate (NHP, Callithrix jacchus) or fixed Vero cells, actively replicating a first passage contemporaneous strain [19] six days post infection, were used as positive controls (vi). To control for non-specific background labeling, parallel placental specimens from Cases 1–4 were imaged after parallel hybridization in the absence of a ZIKV-specific probe (vii).

Due to unavoidable delays and challenges with clinical placental ZIKV testing, including receipt of approval, reliably interpretable clinical results may not be available. For example, with Case 3 there was a >10 day delay in sending the specimen while awaiting approval, which resulted in prolonged exposure to formalin solution without the benefit of being embedded (e.g., not formalin-fixed paraffin embedded (FFPE)) [20]. Anticipating these challenges, subjects were consented for “research only” rapid placental testing, the findings of which are reported here (Figure 4 and Figure 5). 

Results of qualitative and semi-quantitative NAT of flash-frozen placental tissue, amnion-chorion membranes, and transected umbilical cord are shown in Figure 4A,B. As shown in Figure 4A, ZIKV nucleic acid was detected by NAT, and specific to placental specimens from congenital Zika syndrome affected cases (Cases 1, 2, and 3) but not to the unaffected case (Case 4). This was not incidental detection of contaminating maternal NAT viremia, since both Case 1 (congenital Zika syndrome affected) and Case 4 (unaffected) had seroconverted to negative two weeks (Case 1) or 15 weeks (Case 4) prior to delivery (Figure 1). Moreover, qualitative placental, cord and chorion/amnion membranous NAT (Figure 4A) was observed in two instances of congenital Zika syndrome with no evidence of maternal viremia nor viruria nor positive IgM serology (Case 2 and 3; Figure 1). Quantitation of relative measures of ZIKV RNA by NAT (74 nt) demonstrated significantly greater amounts of ZIKV RNA in human placental tissue among congenital Zika syndrome affected (Cases 1–3; Figure 4B) relative to the unaffected (Case 4) pregnancy. The diagnosis of congenital Zika syndrome was highly unlikely to be an undiagnosed rare genetic condition, as all affected cases demonstrated summarily normal genomic variants without evidence of chromosomal structural variations nor pathogenic SNPs on CMA (Figure 4C). Case 2 did harbor a novel de novo heterozygous VUS in the *MED12* gene, however the phenotypic findings in this female infant was most consistent with congenital Zika syndrome. 

In order to localize and visualize stable and actively replicating placental ZIKV in situ, several independent microscopy assessments were employed. These included labeling of *Flaviviridae* glycoprotein E with the 4G2 monoclonal antibody (Figure 5A), and RNA ISH against both (+) and (−) ZIKV-specific ssRNA (Figure 5B,C, respectively). Independent histologic examination of the placental villous tree and parenchyma were without evidence of inflammation nor significant pathologic changes in any of the four cases (Figure 5D). Key findings of these microscopy experiments include: (i) Evidence of flavivirus glycoprotein E by 4G2 positive antibody staining in two of three congenital Zika syndrome-affected infants placentae, but not in the unaffected case (Sase 4); (ii) among all congenital Zika syndrome affected cases, stable and active ZIKV production in the placental cells (presumptive macrophages and trophoblasts, including multinucleated presumptive syncytiotrophoblasts) *per se*, as measured by highly sensitive stably amplified ISH with ZIKV probes against both the (+) and (−) RNA strands; (iii) absence of placental inflammation nor significant ZIKV-associated pathologic changes in all cases.

## 3. Discussion

Although limited by a small sample size, the main findings in our series suggest a concordance between congenital Zika syndrome and detection of ZIKV replication in freshly analyzed placental tissue and its cells (largely trophoblasts and macrophages). In this study we went beyond current standard testing, and used the following to demonstrate placental ZIKV replication: (1) Flavivirus glycoprotein E (4G2 antibody labeling); (2) NAT, including quantitative and qualitative abundance measures; and (3) ZIKV (+) and (−) ssRNA labeling by in situ hybridization. In each of these illustrative cases, due to probable delayed maternal testing relative to actual infection, prenatal detection either waned (Cases 1 and 4) or was negative (Cases 2 and 3), thus demonstrating the limitations of current serologic and molecular testing for congenital Zika syndrome. 

Our study is not without limitations. By design, our study is limited to four recent cases and; therefore, may not represent testing and findings in the population at-large. However, our cases and their characteristics are examples of current challenges in obstetrical care, which include care of emigrant gravidae from Zika-endemic regions whose timing of infection cannot be reliably determined. Accordingly, maternal and newborn testing either reverted to negative (Cases 1 and 4) or was never positive (Cases 2 and 3), and was summarily non-diagnostic of congenital Zika syndrome. Because we were aware of the limitations to interpretation of negative maternal ZIKV laboratory testing in the setting of prolonged endemic exposure, when we encountered concerning findings on prenatal sonogram and MRI, our differential diagnoses continued to include congenital Zika syndrome. Because placental ZIKV testing occurred post-delivery and was under research auspices only, our findings did not guide our clinical care, management, nor eventual postnatal consensus diagnosis of congenital Zika syndrome.

Despite these limitations, our clinical and experimental characterization of these four cases illustrate several relevant findings, which meaningfully extend prior observations. In a recent series from the CDC [25], among 81 pregnancy losses submitted for FFPE-based placental testing, 18 (22%) were positive by ZIKV RT-PCR and nine out of 86 (10%) of live births with negative maternal serologies were placental ZIKV RT-PCR positive [25]. In this same study, among an expanded cohort of 627 completed pregnancies, 33 live births were reported to have possible Zika-virus-associated birth defects. Sixteen of these 33 (53%) presumptive congenital Zika syndrome cases were placental FFPE-ZIKV RT-PCR positive, but there was no capacity to distinguish between maternal and congenital infection. Other observational studies have similarly been challenged to either distinguish or explain discordant maternal testing with evident congenital Zika syndrome disease (18–24). Because we utilized (+) and (−) RNA strand ISH, we can localize areas of ongoing viral RNA replication in placental tissue and cells (Figure 5B,C), and distinguish from maternal blood contamination. We specifically localized both (+) strand and (−) strand ZIKV replication to the villous and stromal placental cells, which harbor both trophoblasts (cytotrophoblasts and multinucleated syncytiotrophoblasts) and placental villous stromal macrophages (Hofbauer cells). We further found ZIKV (as measured by RT-PCR NAT) in not just primary human placental cells at term, but the epithelial and endothelial cells of the amniochorion and umbilical cord (Figure 4). These data collectively provide in situ evidence for ongoing transplacental and paraplacental replication, consistent with reservoirs and routes for fetal infection months after maternal clearing of the virus in the serum and urine, and loss of IgM seropositivity (Figure 1).

These observations pertaining to the role of the placenta, as both maternal-fetal ZIKV conduit and reservoir, are consistent with ours and other in vitro human and in vivo animal model studies (25–31; 37–39). Specifically, use of experimentally manipulated rodent congenital Zika syndrome models and human primary trophoblast infectivity studies have shown that trophoblasts are in fact permissive for ongoing low-level ZIKV replication [24,26,27,28,37,38], and villous stromal Hofbauer cells undergo proliferation and hyperplasia with active viral replication [27,28,29,30,31]. Ours and others observations are in contrast to an initial report by Bayer et al. [32] suggesting trophoblast resistance to ZIKV infection.

Appreciation of the role of the placenta in congenital infections is important for both understanding pathogenesis and preventing harm from current and emerging infectious diseases. Classically, the placenta has been considered to be an effective maternal–fetal barrier. However, a more holistic view, which incorporates the importance of intrauterine exposure to commensal microbes and the need for maternal–fetal dialogue during development, alternately suggests that the placenta functions as an effective conduit and channel of communication between mother and fetus [35,36]. In early gestation, self-renewing trophoblasts from chorionic villi differentiate to cyto- and syncytiotrophoblasts, which anchor the placental chorionic villi to the uterine decidua to channel blood from the circulation after remodeling of spiral arteries [22,28,29]. To maintain immune tolerance to a hemi-allogenic placenta, Natural Killer (NK) and monocyte cell derivatives migrate to the basal decidua. As pregnancy progresses into the second trimester, differentiated invasive cytotrophoblasts will migrate into the opposing parietal decidua to attach to the amniochorionic membranes, thereby enabling the parietal decidua to retain maternal blood and lymphatic vessels and serve as a site of maternal-fetal exchange. Interestingly, Hofbauer cells are villous stromal cells of fetal origin and first appear in the chorionic villi prior to establishment of fetal circulation in the early first trimester [28,29]. Their functions are similar to other tissue macrophages of monocytic origin, including phagocytosis and antigen presentation. In the absence of evidence of active inflammation (which would necessarily result from maternal immune activation in the basal decidua), ZIKV would be permissive to replication in both self-renewing placental trophoblast populations and Hofbauer macrophages across the entirety of gestation. This would allow the placenta to serve as an effective long term “shuttle” to the fetus, functioning first as a reservoir during the early period of immune tolerance and later as a source of virions for fetal infection to susceptible cell types and tissue, including fetal neuroprogenitor cells [19,24,28,29,35,36,37,38]. Our triad of accompanying observations (congenital brain malformations in the absence of placental inflammation, but exclusive presence of ongoing stable and active ZIKV replication in the placenta villi and stroma) is consistent with such a view and our previous research [39].

Our observations have immediate practical implications, as well as longer term translational significance. First, these findings suggest that human placental cells could be used in high throughput screens for potential pharmacologic inhibitors and “druggable” targets. Given the more than four million births annually in the U.S., there is no foreseeable shortage of such currently discarded placental material. We would speculate that identification of drugs that prevent placental infection and viral transport may be critical to prevent the irreversible fetal neuronal damage associated with congenital Zika syndrome. Second, current placental testing is limited to high quality RNA isolated from placenta, and ZIKV replication is measured with PCR primers and probes against the (+) RNA strand. This report demonstrates that molecular diagnostic tools aimed at ZIKV replicon measures, other than ZIKV RT-PCR from FFPE tissue, could be readily designed and deployed, regionally and locally, obviating the need for FFPE fixation, storage, and shipping. Third, our observations serve as a poignant reminder that a “negative” maternal ZIKV test conducted outside of precise and known windows of endemic exposure has marked limitations in its interpretation. Fourth, these observations serve as in situ human evidence that the placenta can serve as a functional reservoir and shuttle for a low-fidelity RNA flavivirus (ZIKV), with molecular evidence of recent functional mutations [24,34].

Given the worldwide prevalence of many other existing and emerging RNA viruses, it is worthwhile and likely imperative to explore their potential for congenital harm in experimental models. In the present case series, diagnosis of congenital Zika syndrome required attentive obstetricians, a working knowledge of the limitations of existing testing, and a honed differential diagnosis when encountering patients from known endemic regions with a characteristic pattern of fetal sonographic findings [19]. It is our hope that this report will prompt reexamination of existing placental specimens and provide additional, measurable diagnostic endpoints in prospective studies, since ZIKV continues to emerge in newly endemic communities in Texas, and other at-risk regions of the Americas and the world.

## 4. Materials and Methods

### 4.1. Study Design and Specimen Collection

*Study design.* The intent of this study was to describe maternal, fetal, neonatal, and infant characteristics among three consecutive subjects within our institution, with a prenatal diagnosis of suspected congenital Zika syndrome but inconclusive testing, and compare that to the next successive subject without suspected congenital Zika syndrome but with early serologic findings. All subjects were enrolled under Baylor College of Medicine IRB H—25735 (most recent approval 5/30/2018). Subjects were randomly assigned Case 1 through 4 designations for the purposes of publication and communication of non-identifying information.

*Subjects.* Subjects were recruited by trained physicians and research study personnel who approached four successive eligible gravidae with either a risk of or suspected congenital Zika syndrome during antenatal care clinic or prior to delivery, from December 2016 to July 2017. At the time of consent, subjects were informed that in addition to reporting their clinical testing, the study would involve a series of “research only” tests of their specimens, including the placenta. There were further informed that even if members of their providing teams of physicians were aware of the results of any “research only” testing, the findings (positive or negative) would not guide clinical care. After consent was obtained, available and relevant clinical information was curated from the electronic medical record and accompanying prenatal records alongside directed subject questioning. All subjects underwent prenatal and postnatal imaging as per contemporaneous clinical recommendations for gravidae at-risk for ZIKV exposure [19].

*Clinical genomic testing.* Chromosomal microarray (CMA) was performed with either Quest or LabCorp Diagnostics platforms, at 1.15 KB resolution with 2.67 million probes (Quest, Houston, TX, USA) or 743K SNP/1.953M NPCN probes (LabCorp Integrated Genetics, Albuquerque, NM, USA) and compared against the GRCH37/hg19 human genome assembly. Whole exome sequencing (WES) was run at Baylor Genetics (Baylor College of Medicine, Houston, TX, USA) as a Critical Trio Whole Exome Sequencing: Proband and included blood specimens from the 6-day old neonate, her mother, and her father (trio).

*Postnatal sample collection.* Immediately following delivery, placentae were removed to a clean dissection lab with sterile surfaces. For histological analysis, a full cross section of placental tissue was removed 4 cm from the cord insertion point and placed in sterile formalin. Tissue was fixed for 8 h before processing into paraffin embedded blocks. Small 1 cm^2^ sections of tissue were trimmed from the chorioamnionic membrane at the site of rupture, and the umbilical cord was transected to yield 3 cm cross-sections of the fetal umbilical cord. For placental specimens designated for NAT, the chorion and amnion layers were dissected for separate analysis, and the underlying fetal chorionic tissue was utilized. All non-formalin-fixed specimens were flash frozen under clean, sterile conditions and stored at −80 C for later molecular analysis.

### 4.2. ZIKV Molecular Testing

*NAT for detection of genomic ZIKV RNA*. In a sterile class IIA biological safety cabinet, approximately 100 mg of frozen membrane, umbilical cord, or placental tissue was used for RNA extraction with TRIzol (Invitrogen). Isolated RNA yield was determined by NanoDrop (Thermo Scientific), and 1 µg converted using a High Capacity cDNA Reverse Transcription Kit (Applied Biosystems). Libraries where then probed for ZIKV genomic sequence using a previously described primer set ZIKV 1086 CCGCTGCCCAACACAAG (fwd), and ZIKV 1162c CCACTAACGTTCTTTTGCAGACAT (rev) [18,19]. Amplicons of contemporaneous strain ZIKV ssRNA genome were generated from dissected sections of placental villi, chorioamnion sections, and full cross-sections of umbilical cord in all cases examined. Extractions of nucleic acid were normalized by RNA concentration. Products were assessed with an Agilent 2100 Bioanalyzer using a Nanochip. Relative product abundance and product size were estimated from chromatograms using built in algorithms of Agilent 2100 Expert v.B2.08 against standards.

*Histology.* Serial sections (4 µm) of whole placental cross-sections were prepared to directly assess for evidence of ZIKV infection by NAT, histologic measures, and direct in situ viral detection via ISH. H&E stained sections were examined by a clinical perinatal and placental pathologist with requisite expertise to assess for leukocyte infiltration and inflammation or other abnormalities. Placental specimens were also probed with 4G2 antibody against flavivirus protein E (Millipore, Burlington, MA, USA) as previously described [24].

An amplified in situ hybridization (ISH) was carried out against the ZIKV viral genome using a ZIKV-specific probe set designed for amplification of signal with branching DNA technologies (RNAscope 2.5 HD Detection Kit, Advanced Cell Diagnostic, Inc., Newark, CA, USA). ZIKV ISH amplification assays have been previously validated in both fetal and placental cells and tissue, and use a series of probe sets that hybridize adjacent pairs, such that adapter probe is proximally associated with enzymatic production in a series of stable amplifications [24,25]. This provides improved specificity and sensitivity over conventional in situ hybridization (ISH). Both the (+) and (−) RNA strands were assessed to determine ongoing permissiveness for replication and active viral replication. Vero cells (Green monkey kidney epithelial) infected with contemporaneous ZIKV strains from similar endemic regions of Central America were used as positive controls [24]. Specifically, and as previously described [24], cells were infected with first passage ZIKV HN16 (GenBank: KX928077.1) isolated from a traveler returning to the U.S. from Honduras in 2016 [24]. Following 6 days of in vitro infection and replication, scraped cells were formalin fixed in a syringe, then paraffin embedded. All sections were developed with DAB and assessed under bright field. Images were captured using a Nikon Eclipse 90i microscope (Nikon Instruments, Melville, NY, USA).
